# Exogenous D-ribose promotes gentamicin treatment of several drug-resistant *Salmonella*

**DOI:** 10.3389/fmicb.2022.1053330

**Published:** 2022-11-07

**Authors:** Yanhong Zhou, Yan Yong, Chunyang Zhu, Heng Yang, Binghu Fang

**Affiliations:** ^1^Guangdong Provincial Key Laboratory of Veterinary Pharmaceutics Development and Safety Evaluation, South China Agricultural University, Guangzhou, China; ^2^National Risk Assessment Laboratory for Antimicrobial Resistance of Animal Original Bacteria, South China Agricultural University, Guangzhou, China; ^3^Guangdong Wens Dahuanong Biotechnology Limited Company, Yun Fu, China

**Keywords:** D-ribose, gentamicin, *Salmonella*, metabolomics, resistance

## Abstract

The metabolic microenvironment of bacteria impacts drug efficacy. However, the metabolic mechanisms of drug-resistant *Salmonella* spp. remain largely unknown. This study characterized the metabolic mechanism of gentamicin-resistant *Salmonella* Choleraesuis and found that D-ribose increased the gentamicin-mediated killing of this bacteria. Non-targeted metabolomics of homologous gentamicin-susceptible *Salmonella* Choleraesuis (SCH-S) and gentamicin-resistant *S.* Choleraesuis (SCH-R) was performed using UHPLC-Q-TOF MS. The metabolic signature of SCH-R included disrupted central carbon metabolism and energy metabolism, along with dysregulated amino acid and nucleotide metabolism, vitamin and cofactor metabolism, and fatty acid synthesis. D-ribose, the most suppressed metabolite in SCH-R, was shown to strengthen gentamicin efficacy against SCH-R and a clinically isolated multidrug-resistant strain. This metabolite had a similar impact on *Salmonella.* Derby and *Salmonella.* Typhimurium. D-ribose activates central carbon metabolism including glycolysis, the pentose phosphate pathway (PPP), and the tricarboxylic acid cycle (TCA cycle), increases the abundance of NADH, polarizes the electron transport chain (ETC), and elevates the proton motive force (PMF) of cells, and induces drug uptake and cell death. These findings suggest that central carbon metabolism plays a critical role in the acquisition of gentamicin resistance by *Salmonella*, and that D-ribose may serve as an antibiotic adjuvant for gentamicin treatment of resistant bacterial infections.

## Introduction

*Salmonella* spp. is a highly prevalent foodborne pathogen that poses a serious threat to human and animal health, causing >10 million global infections each year (CDC, 2019). The World Health Organization (WHO) has defined *Salmonella* spp. as a “priority pathogen”(WHO, 2017). To date, >2,500 serotypes have been identified (Hendriksen et al., 2011). One of these, *Salmonella* Choleraesuis (*S.* Choleraesuis), is host adaptive, primarily infecting pigs, causing paratyphoid fever in piglets, and occasionally infecting and causing bacteremia in humans (Chiu et al., 2004; Yang et al., 2012). Human infection most often occurs through exposure to contaminated food or water (Mahon and Fields, 2016). *S.* Choleraesuis is a widely disseminated serotype (Chiu et al., 2004) and is the most common cause of nontyphoid *Salmonella* infection in Thailand (Luk-In et al., 2018). In Europe, the growing number of *S.* Choleraesuis infections among wild boars has attracted research attention (Longo et al., 2019; Papic et al., 2021; Ernholm et al., 2022). In humans, this pathogen has higher invasiveness and is thus a greater health threat than other serotypes (Cohen et al., 1987).

Antibiotics play a critical role in the treatment of bacterial infections. However, antimicrobial resistance caused by improper antibiotic use is becoming a serious threat (Bush et al., 2011), particularly with the emergence of multidrug-resistant strains (Hogberg et al., 2010; Ciorba et al., 2015). In addition to methicillin-resistant *Staphylococcus aureus* (MRSA; Otto, 2013), extended-spectrum β-Lactamase producing *Enterobacteriaceae* (ESBLs; Castanheira et al., 2021), and vancomycin-resistant *Enterococcus* (VRE; Reyes et al., 2016), multidrug-resistant *Salmonella* is also a serious health problem (Tyson et al., 2021). Specifically, multidrug-resistant *S.* Choleraesuis is becoming a crisis for both humans and animals (Chang et al., 2005; Gil Molino et al., 2019). Resistant strains are non-responsive to all antimicrobial agents, including β-lactams, aminoglycosides, bolome reprogramming by metabsulfonamides (Molino et al., 2020). *Mcr-3.1* was also discovered in *S. Choleraesuis*, rendering colistin ineffective (Oransathid et al., 2022). Unfortunately, the development of more complex mechanisms of resistance and the high cost of creating new agents have hindered effective treatment (Breijyeh et al., 2020; Provenzani et al., 2020). Some studies predict that antimicrobial-resistant pathogens will be attributed to 10 million deaths worldwide by 2050 (Castro-Vargas et al., 2020). Thus, there is an urgent need to stop the emergence and spread of antibiotic resistance.

The metabolic state of bacteria affects their susceptibility to antibiotics (Stokes et al., 2019). Bacterial cells embedded in biofilms are particularly tough to eliminate due to the impermeability of biofilms and the reduced metabolic status of the embedded cells (Yin et al., 2019). Persister cells are subpopulations of antibiotic-sensitive bacteria which escape antibiotic killing through metabolic repression (Fisher et al., 2017). In recent years, new strategies have been developed to explore the metabolic mechanisms of bacterial acquired resistance. Metabolomics is the large-scale evaluation of small molecules in cells or tissues which cannot be studied using other methods, including genomics, transcriptomics, and proteomics (Castro-Santos et al., 2015; Johnson et al., 2016). An important feature of metabolomics is that basic metabolic pathways and metabolites are similar between species, while genes, transcripts, and proteins are species specific (Kovac et al., 2013). Hence, metabolomics is a potential tool to explore the acquisition of antimicrobial resistance by bacteria (Kok et al., 2022). A GC–MS-based metabolomics study on cefoperazone sulbactam (SCF)-resistant *P. aeruginosa* (PA-R_SCF_) found that the metabolic mechanism for resistance was the inhibition of central carbon metabolism (Chen et al., 2022). Another metabolomics study on *Escherichia coli* carrying the *mcr-1* gene found that *mcr-1*-mediated colistin resistance was associated with a disruption in glycerophospholipid metabolism and LPS biosynthesis as well as the accumulation of the substrate PEA (Li et al., 2019). *Mcr-1*-mediated colistin-resistant *Vibrio alginolyticus*, however, showed reduced central carbon metabolism and membrane potential (Li et al., 2020). These differences may be explained by interspecies differences. While the metabolic mechanism of antibiotic resistant *Edwardsiella tarda* and *Campylobacter jejuni* have also been studied (Cheng et al., 2017; Liu et al., 2019), there are few reports on the metabolic mechanism of antibiotic resistance in *Salmonella* spp.

Aminoglycosides, a class of antibiotics that act by inhibiting protein synthesis, are powerful weapons against multiple pathogens, including *Salmonella* (Poulikakos and Falagas, 2013). Unfortunately, several types of bacteria exhibit resistance to aminoglycosides through enzymatic modifications, efflux pump systems, and genetic mutations that threaten their sustainable utilization (Touati, 2019). Strategies are urgently needed to retain these drugs in the antibacterial arsenal (Bottger and Crich, 2020). Metabolome reprogramming by metabolites is an effective strategy against drug resistance (Peng et al., 2015a). For example, alanine, glucose, and fructose can promote kanamycin-mediated killing of *Edwardsiella tarda*. These metabolites induce NADH through the TCA cycle, which activates the proton motive force (PMF) and enables drug uptake (Peng et al., 2015b). A recent study also reported that glutamine promotes the function of antibiotics against multidrug-resistant bacteria (Zhao et al., 2021). This study analyzed the metabolic spectrum of clinically isolated multidrug-resistant and susceptible *Escherichia coli* and identified glutamine, which was inhibited by drug-resistant bacteria, as a biomarker. Exogenous glutamine promoted the β-lactams-, aminoglycosides-, quinolones- and tetracyclines-induced killing of multidrug-resistant uropathogenic bacteria and enhanced the activity of ampicillin against multidrug-resistant *Klebsiella pneumoniae*, *Pseudomonas aeruginosa*, *Acinetobacter baumannii*, *Edwardsiella tarda*, *Vibrio alginolyticus*, and *Vibrio parahaemolyticus*. These results indicate that metabolome reprogramming may be an effective weapon against drug resistance. The current study sought to investigate whether similar mechanisms can be used to improve the activity of other aminoglycosides, including gentamicin, tobramycin, apramycin, and kanamycin, against antibiotic-resistant *Salmonella* spp.

UHPLC-Q-TOF MS was used to characterize the metabolic profile of gentamicin-susceptible and resistant *S.* Choleraesuis and explore the metabolic mechanisms of gentamicin resistance of SCH-R. Resistance was associated with a disruption in central carbon metabolism, along with dysregulated amino acid and nucleotide metabolism, vitamin and cofactor metabolism, and fatty acid synthesis. In addition, D-ribose, the most suppressed metabolite in SCH-R, significantly increased the gentamicin-mediated killing of both SCH-R and the wild strain and had a similar effect on multidrug-resistant *Salmonella*. These findings provide new perspectives and strategies for the study and resolution of *Salmonella* resistance mechanisms.

## Materials and methods

### Chemicals

All antibiotics were purchased from the China Institute of Veterinary Drug Control (Beijing, China). Mueller Hinton (MH) Agar, MH Broth, Tryptic Soy Agar (TSA), Luria-Bertani (LB) broth, MacConkey Agar, and Xylose Lysine Deoxycholate (XLD) Agar were purchased from Guangdong Huankai Microbial Sci & Tech. Co., Ltd. (Guangdong, China). Methanol and acetonitrile (high-performance liquid chromatography grade) were purchased from Thermo Fisher Scientific (Waltham, MA, United States). Ammonium ethoxide and formic acid (high-performance liquid chromatography grade) were purchased from Sigma Aldrich (Missouri, United States).

### Bacterial strains and growth conditions

Standard strains of *Escherichia coli* (ATCC25922) and *S.* Choleraesuis (ATCC13312) were purchased from American Type Culture Collection (Manassas, VA, United States). The clinical strains, SR-1 and SR-6 (*S.* Derby), SR-7 (*S.* Typhimurium), and SCH2021 (*S.* Choleraesuis) were isolated from swine farms in South China (Guangdong Province, China). These strains exhibit resistance to most clinical antimicrobials, including β-lactams, aminoglycosides, tetracyclines, and sulfonamides. The minimum inhibitory concentrations (MICs) of antimicrobials against different *Salmonella* strains were determined using the microdilution method (Li et al., 2020). The standard *S.* Choleraesuis (ATCC13312) strain was sequentially propagated with or without drug and drug-resistant bacteria were selected. All bacterial cultures were grown in LB medium at 37°C.

### Sample pretreatment

*S.* Choleraesuis (ATCC13312) was used for non-targeted metabolomics studies. Gentamicin-resistant (SCH-R), and susceptible (SCH-S) bacteria were cultured in LB broth to a late exponential phase. The bacterial suspension (5 ml) was centrifuged at 16,000 g for 10 min at −10°C and the precipitate was washed twice with PBS. The bacteria were collected and quickly quenched in liquid nitrogen to halt metabolism. Methanol/acetonitrile/water (1 ml, 2:2:1, V/V) was added, vortexed for 60 s, and extracted ultrasonically twice at a low temperature. The supernatant was placed at −20°C for 1 h to precipitate the proteins. The samples were centrifuged at 20,000 g at 4°C for 20 min, and the supernatant was collected, lyophilized, and stored at −80°C.

### Non-targeted metabolomic analysis

UHPLC-Q-TOF MS was used to perform the non-targeted metabolomics analysis. Samples were separated on a HILIC column at 25°C with mobile phase A (water +25 mM ammonium acetate +25 mM ammonia) and mobile phase B (acetonitrile). The gradient elution procedure included: 0–1 min, 95% B; 1–14 min, 95–65% B; 14–16 min, 65–40% B; 16–18 min, 40% B; 18–18.1 min, 40–95% B; 18.1–23 min, 95% B. The flow rate was 0.3 ml/min with an injection volume of 2 μl. Electrospray ionization (ESI) was used to monitor the samples in positive and negative ion modes using the following parameters: Ion Source Gas 1 (Gas1) = 60, Ion Source Gas 2 (Gas 2) = 60, Curtain gas (CUR) = 30, source temperature = 600°C, IonSapary Voltage Floating (ISVF) = ± 5,500 V; TOF MS scan m/z range = 60–1,000 Da, product ion scan m/z range = 25–1,000 Da, TOF MS scan accumulation time = 0.20 s/spectra, and product ion scan accumulation time = 0.05 s/spectra. The secondary mass spectrum was obtained by information-dependent acquisition (IDA) and used with a high sensitivity mode as follows: Declustering potential (DP) = ± 60 V (positive and negative modes), collaboration energy = 35 ± 15 EV. IDA was set to exclude isotopes within 4 Da and monitor six candidate ions per cycle. Quality control (QC) samples were inserted into the sample queue to monitor and evaluate the stability of the system and ensure data reliability.

### Data processing and statistical analysis

Mass spectrometry data were processed using Software Analyst 1.6. The total ion and MRM multi-peak diagrams of the samples were obtained. The mass spectrum peaks detected by each metabolite in different samples were corrected based on the retention time and peak metabolite types. Principal component analysis (PCA), partial least squares discrimination analysis (PLS-DA), orthogonal partial least squares analysis (OPLS-DA), multivariate analysis, and the Student’s t-test were used to investigate intra- and inter-group differences between the samples and to identify differential metabolites. Using OPLS-DA analysis, differences between groups were preliminarily screened using a Variable Importance in Projection (VIP) score > 1. Univariate statistical analysis was used to verify whether the differential metabolites were significant. Those with multidimensional statistical analysis VIP scores >1 and a univariate statistical analysis *p* value of <0.05 were selected as significantly different. The structure of each metabolite was identified using accurate mass number matching (<25 ppm) and secondary spectrum matching. To comprehensively display the relationship between samples and the differences in the expression patterns of metabolites in different samples, we perform hierarchical clustering on each group of samples to help us accurately screen marker metabolites. Finally, KEGG pathway analysis and pathway enrichment of differential metabolites were carried out to identify those metabolic pathways with the most significant changes.

### Antibiotic bactericidal assays

The antibacterial assay was carried out as described previously (Allison et al., 2011). In brief, bacterial cells were cultured in LB broth to the late exponential phase. After centrifugation at 10,000 g for 5 min, the cells were washed twice with sterile PBS buffer and resuspended to 1 × 10^6^ cfu/ml in M9 minimal media. D-ribose and antibiotics were added, and the culture was incubated at 37°C for 6 h. In all CCCP experiments, the cells were preincubated with 20 μM CCCP for 5 min before adding metabolites or antibiotics. The remaining inhibitors, bromopyruvate, malonate, and rotenone, were added at the same time as the metabolites or antibiotics. After incubation, 100 μl of the culture was removed, serially diluted, and plated (20 μl aliquots) onto LB agar plates. The agar plates were incubated at 37°C for 10–16 h and those with 10–100 colonies were enumerated to determine the number of colony-forming units (cfu). Percent survival was determined by the ratio of cfu obtained from the test and control samples.

### Measurement of NADH and NADH dehydrogenase 1 (ND1)

NADH production was determined using a NAD+/NADH assay kit (BioAssay Systems, Hayward, CA, United States; Peng et al., 2015b). In brief, bacterial cells were grown to the exponential phase (6 h) in broth, and 1 ml of the culture was collected and centrifuged at 13,000 g for 5 min. The supernatant was discarded, and the pellet was washed three times with PBS. NADH extraction buffer (100 μl) was added, the extract was heated at 60°C for 5 min, then 20 μl assay buffer and 100 μl NAD^+^ extraction buffer were added to neutralize the extract. After centrifugation, NADH was measured in the supernatant according to the manufacturer’s instructions. NADH dehydrogenase 1 was assessed using a Microorganism ND1 ELISA kit (Jiangsu MEIMIAN Industrial Co., Ltd., Yancheng China). The exponential phase culture (1 ml) was centrifuged and washed, and the cells were resuspended in 1 ml of PBS. The cells were then disrupted by sonication to obtain a supernatant. ND1 was detected according to the manufacturer’s instructions.

### Measurement of membrane potential

The membrane potential was examined using a *BacLight* Bacterial Membrane Potential Kit (Life Technologies, Carlsbad, CA, United Statessss) as previously described (Yong et al., 2021).

### Measurement of intracellular gentamicin

Measurement of intracellular gentamicin was determined as previously described (Yong et al., 2021). In brief, bacterial cells were cultured in LB broth until the late exponential phase. After centrifugation, the precipitates were collected and resuspended in 10 ml of M9 minimal media to 1 × 10^6^ cfu/ml with or without antibiotics and D-ribose. The reaction samples were incubated at 37°C for 6 h, and the precipitates were harvested by centrifugation, washed twice with PBS, and resuspended in 1 ml of PBS. The solution was sonicated for 10 min and the insoluble substances were removed by centrifugation. Gentamicin was quantified in the supernatant using a Gentamicin ELISA kit (Shanghai Enzyme-linked Biotechnology Co., Ltd., Shanghai, China).

### Quantitative RT-PCR analysis

Bacterial cells were harvested in the exponential phase. The total RNA was extracted according to the manufacturer’s instructions using an RNAiso Plus kit (Takara Japan). Reverse transcription-PCR was performed using a Hifair® II 1st Strand cDNA Synthesis Kit (Yeasen, Shanghai China) with 1.5 μg of total RNA according to the manufacturer’s instructions. QRT-PCR was performed with a total volume of 10 μl liquid including 5 μl 1x SYBR Green Master Mix (Yeasen, Shanghai China), 1 μl PCR-grade water, 2 μl cDNA template, and 1 μl of each primer pair (0.2 μM) using a real-time PCR system (BIO-RAD). The specific primers are listed in the Supplementary material (Supplementary Table 1). The cycling parameters were as follows: pre-denaturation at 95°C for 300 s, 40 cycles of 95°C for 10 s, and 60°C for 30 s. The melting curve was obtained from 60–95°C with a calefactive velocity of 0.05°C/s. Differences in gene expression were compared between the experimental and control groups and 16S rRNA was used as the internal reference. The biological repeats were carried out in triplicate.

## Results

### Metabolic profile of SCH-R

**Figure 1:**
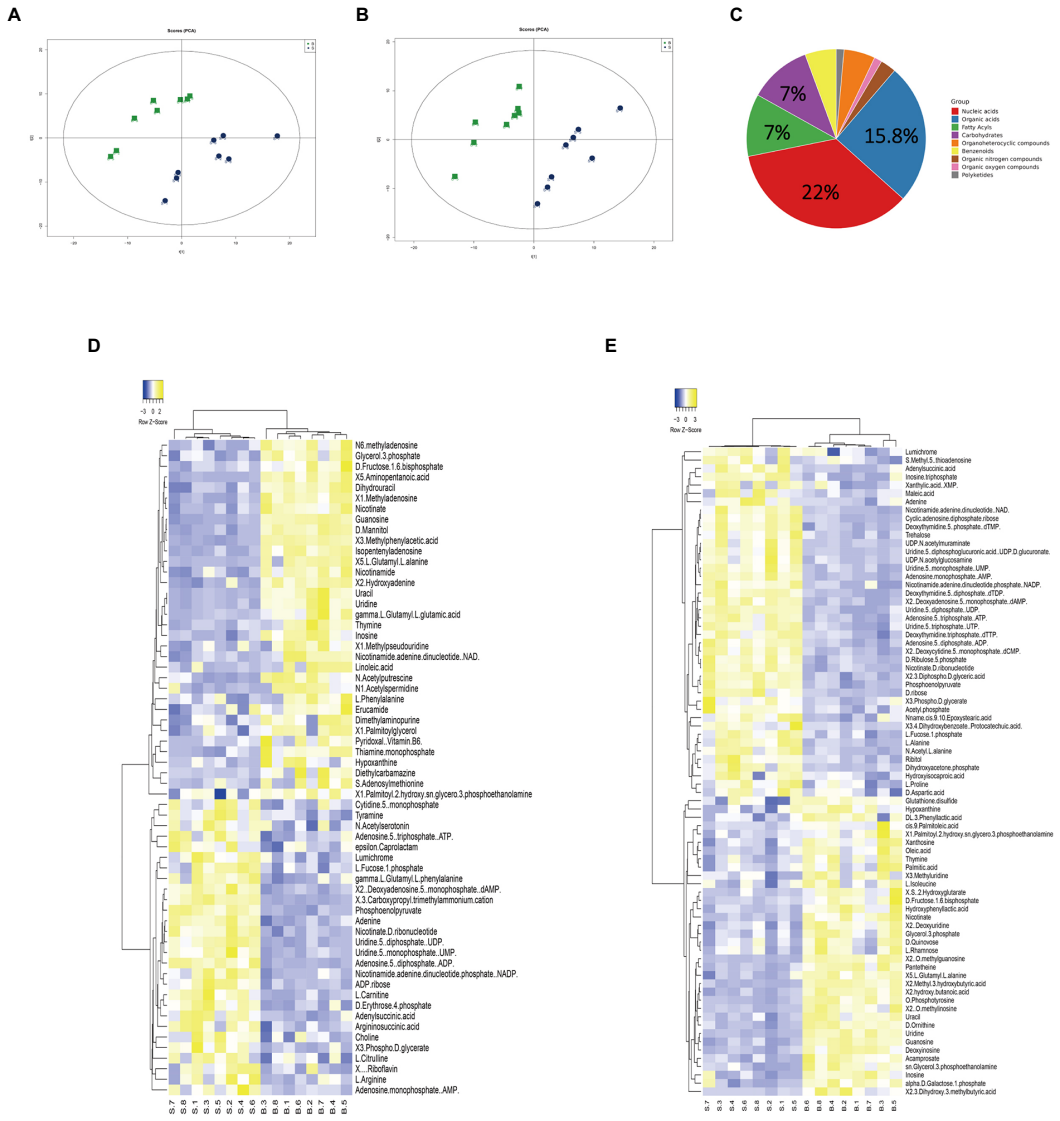
Analysis of the SCH-R metabolic profile. **(A)** PCA score of gentamicin-resistant and sensitive *Salmonella* in positive ESI modes. **(B)** PCA score of gentamicin-resistant and sensitive *Salmonella* in negative ESI modes. **(C)** Abundance of different categories of metabolites in SCH-R. **(D)** Cluster analysis of different metabolites in positive ESI modes. **(E)** Cluster analysis of different metabolites in negative ESI modes. “B” represents gentamicin-induced resistant *Salmonella* ATCC13312, “S” represents gentamicin-sensitive *Salmonella* ATCC13312.

Gentamicin-susceptible *S.* Choleraesuis (ATCC13312, SCH-S) was continuously cultured in LB broth with or without gentamicin to obtain gentamicin-resistant *Salmonella* (SCH-R), and microdilution method was used to detect the MIC of different antimicrobials against SCH-R and clinical isolates (Table 1). A non-targeted metabolomics profile of SCH-S and SCH-R was then determined using UHPLC-Q-TOF MS with eight biological replicates per group. Unsupervised PCA was performed on the ion peaks extracted from each group of samples to obtain the overall difference profile between the groups (Figures 1A,B). To better reflect the differences between samples, OPLS-DA analysis was performed, and the VIP score was used to help identify differential metabolites. Univariate analysis was also conducted to ensure the reliability of differential metabolites, and those with a VIP score > 1 and a value of *p* <0.05 were considered significant. A total of 114 differential metabolites were identified in the SCH-S and SCH-R strains in the positive and negative ion modes after the same species were removed, of which 59 were up-regulated and 55 were down-regulated (Supplementary Table 2). The four most abundant of the 114 metabolites were nucleic acids (22%), organic acids (15.8%), carbohydrates (7%), and fatty acyls (7%) (Figure 1C). Hierarchical clustering of differential metabolites was used to show the relationship between samples and help to accurately identify biomarkers with significant significance (Figures 1D,E). These results indicated that the metabolic state of SCH-R was disrupted.

**Table 1:** MIC value of different antimicrobials against *Salmonella* (μg/mL).

	**ATCC13312S (SCH-S)**	**ATCC13312R (SCH-R)**	**SCH2021**	**SR-1**	**SR-6**	**SR-7**
Gentamicin	0.25	16	16	32	16	32
Tobramycin	0.25	0.25	64	32	32	32
Apramycin	2	2	64	128	256	256
Kanamycin	2	2	256	512	512	512
Spectinomycin	8	8	256	512	512	512
Ampicillin	2	2	32	128	32	32
Amoxicillin/Clavulanic acid	1	1	32	32	32	16
Ceftiofur	0.5	0.5	2	1	0.5	0.5
Ofloxacin	0.03	0.03	4	8	4	8
Enrofloxacin	0.03	0.03	4	4	4	4
Tetracycline	1	1	32	32	16	16
Colistin	0.5	0.5	1	1	1	1
Sulfaisoxazole	4	4	512	512	512	512
Sulfamethoxazole/Trimethoprim	0.5	0.5	32	32	32	32

### Enriched pathways in SCH-R

**Figure 2:**
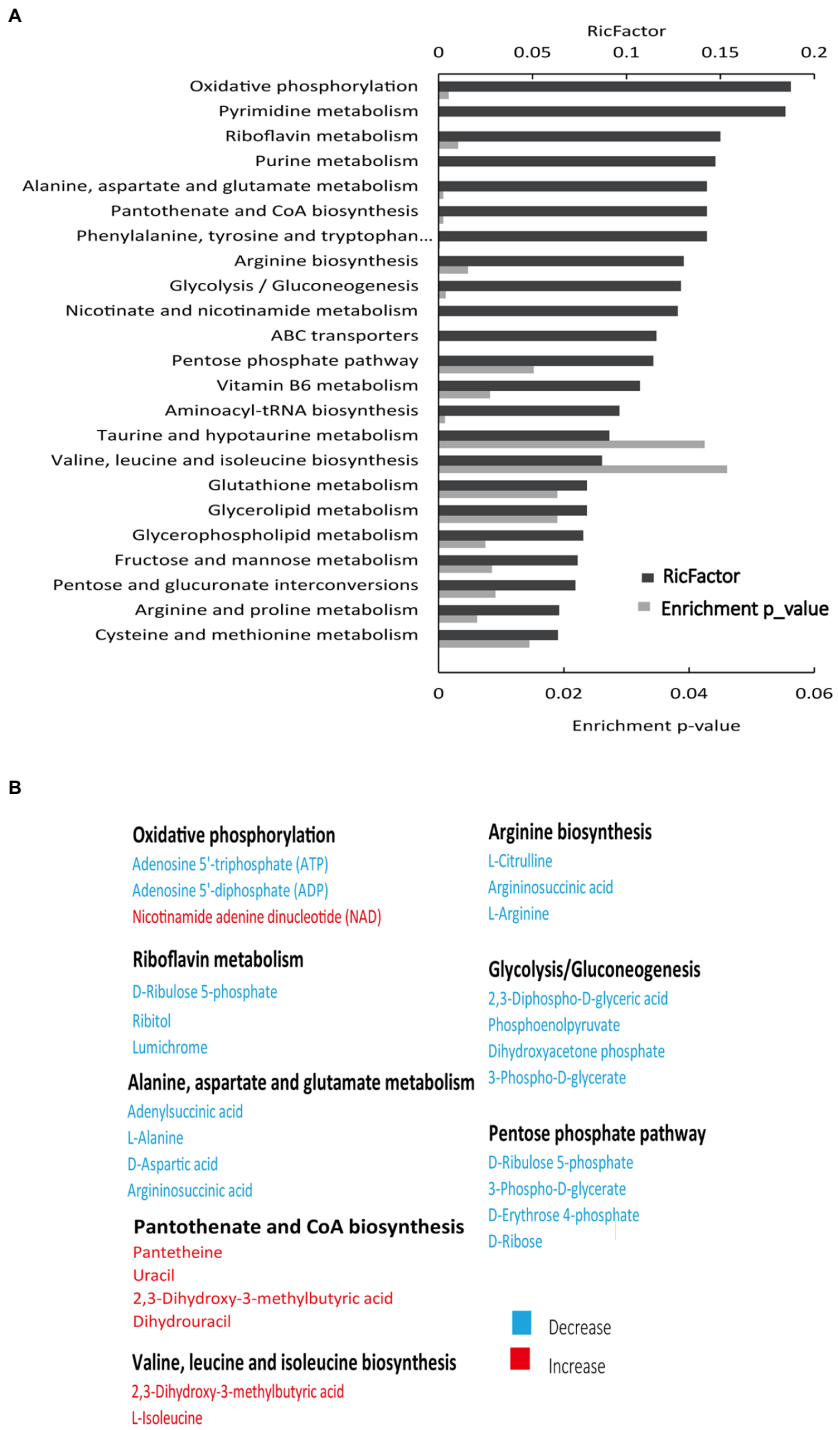
Enriched pathways in SCH-R. **(A)** Pathway enrichment of metabolites in SCH-R and SCH-S. **(B)** Differential expression of metabolites from important metabolic pathways. Red color indicates increased metabolite levels; Blue color indicates decreased metabolite levels.

To assess the metabolic changes of the drug-resistant bacteria, alterations in metabolite abundance were analyzed using KEGG (https://www.genome.jp/kegg). As shown in Figure 2A, 23 pathways were enriched (Supplementary Table 3). The most impacted pathway was oxidative phosphorylation, a series of chemical reactions occurring in the electron transport chain (ETC) (Bald et al., 2017). PMF generated by ETC is shown to promote the internalization of aminoglycosides (Taber et al., 1987). Thus, we speculated that the PMF of SCH-R was suppressed as a result of disturbed oxidative phosphorylation, ultimately mediating the resistance of SCH-R to gentamicin. The increased abundance of NAD^+^ in SCH-R cells was also suggestive of PMF suppression (Figure 2B). Key metabolites associated with riboflavin metabolism, alanine, aspartate and glutamate metabolism, arginine biosynthesis, glycolysis/gluconeogenesis, and pentose phosphate pathway were down-regulated, while those associated with pantothenate and CoA biosynthesis and valine, leucine, and isoleucine biosynthesis were up-regulated (Figure 2B). Of these, the glycolysis/gluconeogenesis and pentose phosphate pathway are involved in central carbon metabolism, which converts nutrients into biomass and energy (Ryan et al., 2021). The central carbon metabolism provided protons through NADH reduction to maintain PMF. Thus, we reasoned that a disturbed central carbon metabolism in SCH-R affected oxidative phosphorylation and thereby suppressed PMF. Additionally. amino acid metabolism fuels central carbon metabolism, which benefits riboflavin metabolism, fatty acid synthesis, and other pathways. These findings suggested that the global metabolism of SCH-R was altered and that disrupted central carbon metabolism may play a key role in mediating SCH-R resistance.

### Perturbed central carbon metabolism is associated with lower PMF in SCH-R

**Figure 3:**
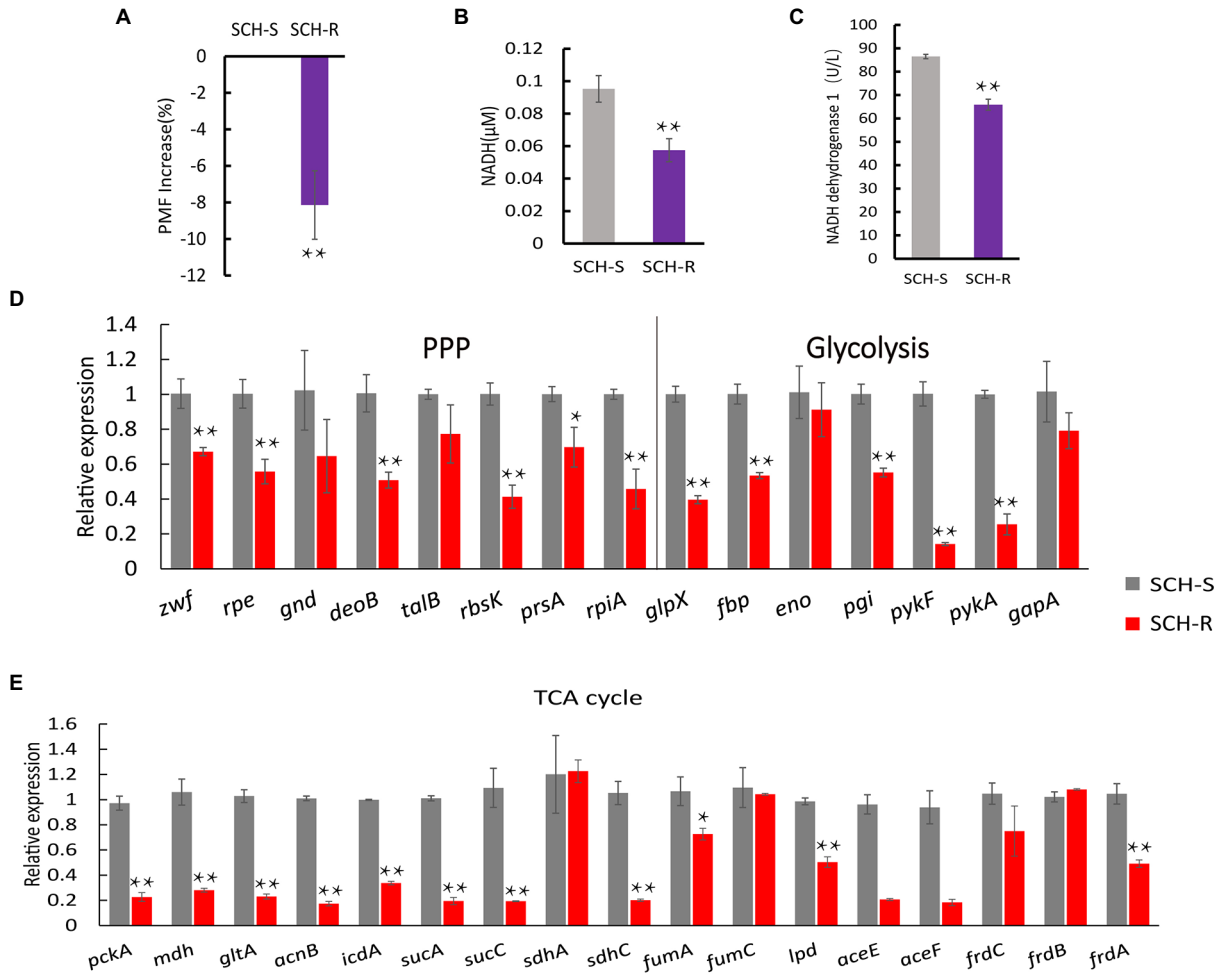
SCH-R resistance is mediated by disrupted central carbon metabolism. **(A)** Variation of the PMF in SCH-R and SCH-S. **(B)** NADH concentration in SCH-R and SCH-S. **(C)** Activity of NADH dehydrogenase 1 in SCH-R and SCH-S. **(D,E)** QRT-PCR for the expression of key genes in central carbon metabolism. *pckA*, phosphoenolpyruvate carboxykinase; *mdh*, malate dehydrogenase; *gltA*, citrate synthase; *acnB*, aconitate hydratase 2; *icdA*, isocitrate dehydrogenase; *sucA*, 2-oxoglutarate dehydrogenase E1 component; *sucC*, succinyl-CoA synthetase beta subunit; *sdhA*, succinate dehydrogenase; *sdhC*, succinate dehydrogenase; *fumA*, fumarate hydratase, class I; *fumC*, fumarate hydratase, class II; *lpd*, dihydrolipoyl dehydrogenase; *aceE*, pyruvate dehydrogenase E1 component; *aceF*, pyruvate dehydrogenase E2 component; *frdC*, fumarate reductase subunit C; *frdB*, fumarate reductase iron–sulfur subunit; *frdA*, fumarate reductase flavoprotein subunit; *zwf*, glucose-6-phosphate dehydrogenase; *rpe*, ribulose-phosphate 3-epimerase; *gnd*, 6-phosphogluconate dehydrogenase; *deoB*, phosphopentomutase; *talB*, transaldolase; *rbsK*, ribokinase; *prsA*, ribose-phosphate pyrophosphokinase; *rpiA*, ribose 5-phosphate isomerase A; *glpX*, fructose-1,6-bisphosphatase II; *fbp*, fructose-1,6-bisphosphatase I; *eno*, phosphopyruvate hydratase; *pgi*, glucose-6-phosphate isomerase; *pykF*, pyruvate kinase; *pykA*, pyruvate kinase; *gapA*, glyceraldehyde 3-phosphate dehydrogenase. Results are displayed as the mean ± SEM and three biological repeats are carried out. Significant differences are identified (*p < 0.05 and **p < 0.01).

To verify whether disturbed central carbon metabolism leads to lower PMF *via* reduced NADH and thus mediates drug resistance, we began by assessing the PMF of SCH-S and SCH-R. Indeed, SCH-R had lower PMF than SCH-S (Figure 3A). NADH, the basis for maintaining PMF, and the activity of NADH dehydrogenase 1 (ND1), were also significantly lower in SCR-R than in SCH-S (Figures 3B,C). These findings suggest that the ETC of SCH-R is severely altered, which leads to lower PMF. To verify whether central carbon metabolism is disrupted, genes involved in glycolysis, PPP, and TCA cycles were assessed by qRT-PCR. As expected, nearly all genes associated with central carbon metabolism were downregulated in SCH-R (Figures 3D,E). These findings suggested that disrupted central carbon metabolism reduced the PMF of SCH-R, potentially accounting for the gentamicin resistance of these bacteria.

### D-ribose induces gentamicin against drug-resistant *Salmonella*

**Figure 4:**
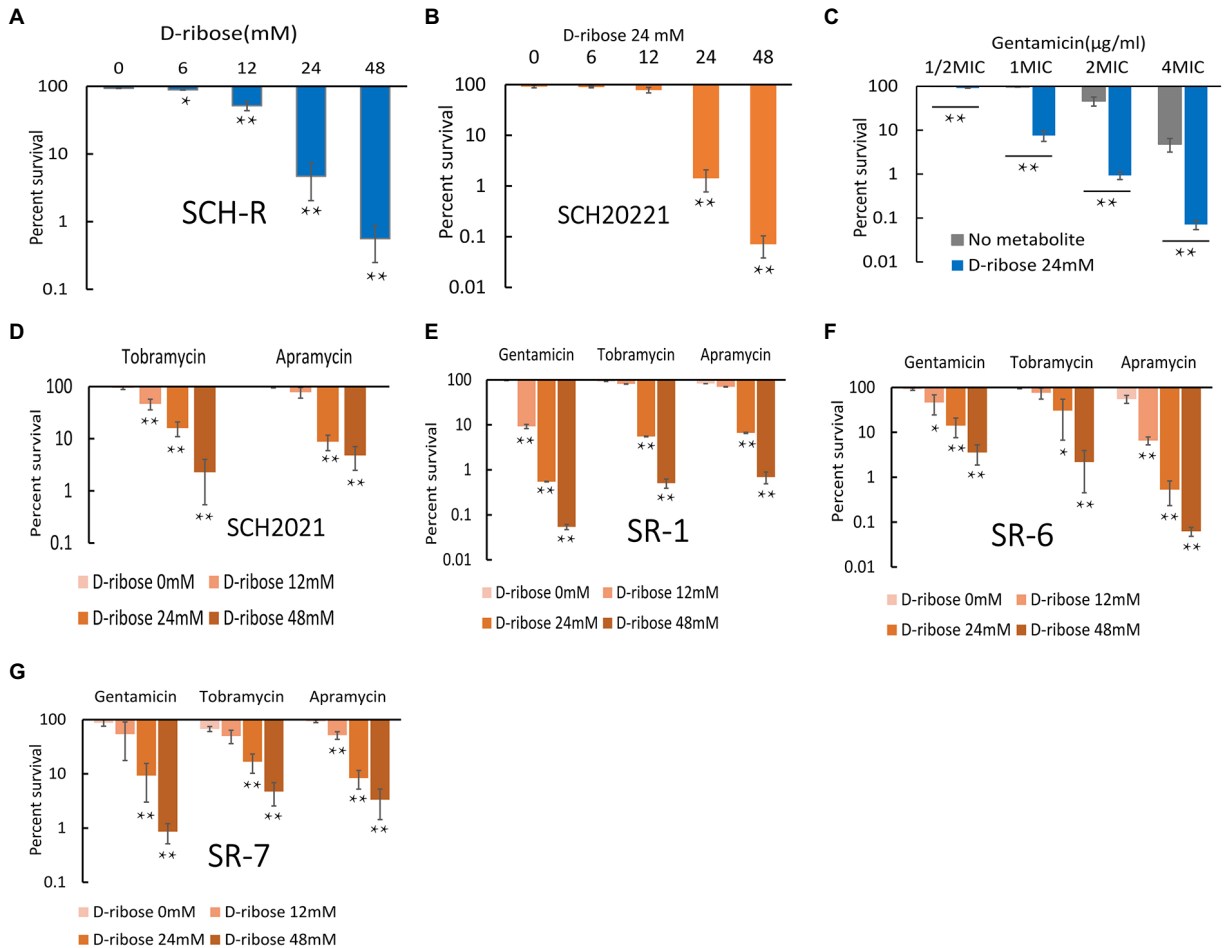
Effect of exogenous D-ribose on SCH-R and clinical isolates of drug-resistant *Salmonella*. **(A)** Percent survival of SCH-R in the presence of 1 MIC gentamicin by D-ribose dose. **(B)** Percent survival of SCH2021 in the presence of 1 MIC gentamicin by D-ribose dose. **(C)** Percent survival of SCH-R in the presence of 24 mM D-ribose by gentamicin dose. **(D–G)** Percent survival of clinical isolates SCH2021 **(D)**, SR-1 **(E)**, SR-6 **(F)**, and SR-7 **(G)** in the presence of different aminoglycosides (1MIC) by D-ribose dose. Results are displayed as the mean ± SEM and three biological repeats are carried out. Significant differences are identified (*p < 0.05 and **p < 0.01).

Exogenous supplementation to reprogram the metabolome by repressed metabolites can alter the sensitivity of resistant strains (Peng et al., 2015b). Our previous study confirmed that citrulline and glutamine promoted apramycin-mediated killing (Yong et al., 2021). The current study confirmed that exogenous D-ribose, which was suppressed in SCH-R, enhanced gentamicin-mediated killing in both SCH-R and SCH2021. SCH2021 is a wild *S.* Choleraesuis which confers resistance to gentamicin, tobramycin, apramycin, kanamycin etc. The two gentamicin-resistant strains were co-incubated with or without D-ribose plus gentamicin. In the presence of gentamicin, cell survival declined with increasing doses of D-ribose (Figure 4A). Specifically, in the presence of 1 MIC gentamicin, when 0, 6, 12, 24, or 48 mM D-ribose was added, the cell survival percentages of SCH-R were 93.46, 88.93, 51.74, 4.69, and 0.56, respectively. Similarly, the cell survival percentages of SCH20221 were 91.50, 89.28, 78.20, 1.41, and 0.071, respectively (Figure 4B). Furthermore, Furthermore, cell survival was gentamicin dose-dependent (Figure 4C; Supplementary Figures S1A–F). D-ribose was also shown to enhance gentamicin-, tobramycin-, and apramycin-mediated killing of multidrug-resistant *S.* Choleraesuis, *S.* Typhimurium and *S*. Derby (Figures 4D–G). These results demonstrated that D-ribose can increase gentamicin-, tobramycin-, and apramycin-mediated killing in drug-resistant *Salmonella*, including multidrug-resistant strains.

### Exogenous D-ribose increases PMF-induced uptake of gentamicin

**Figure 5:**
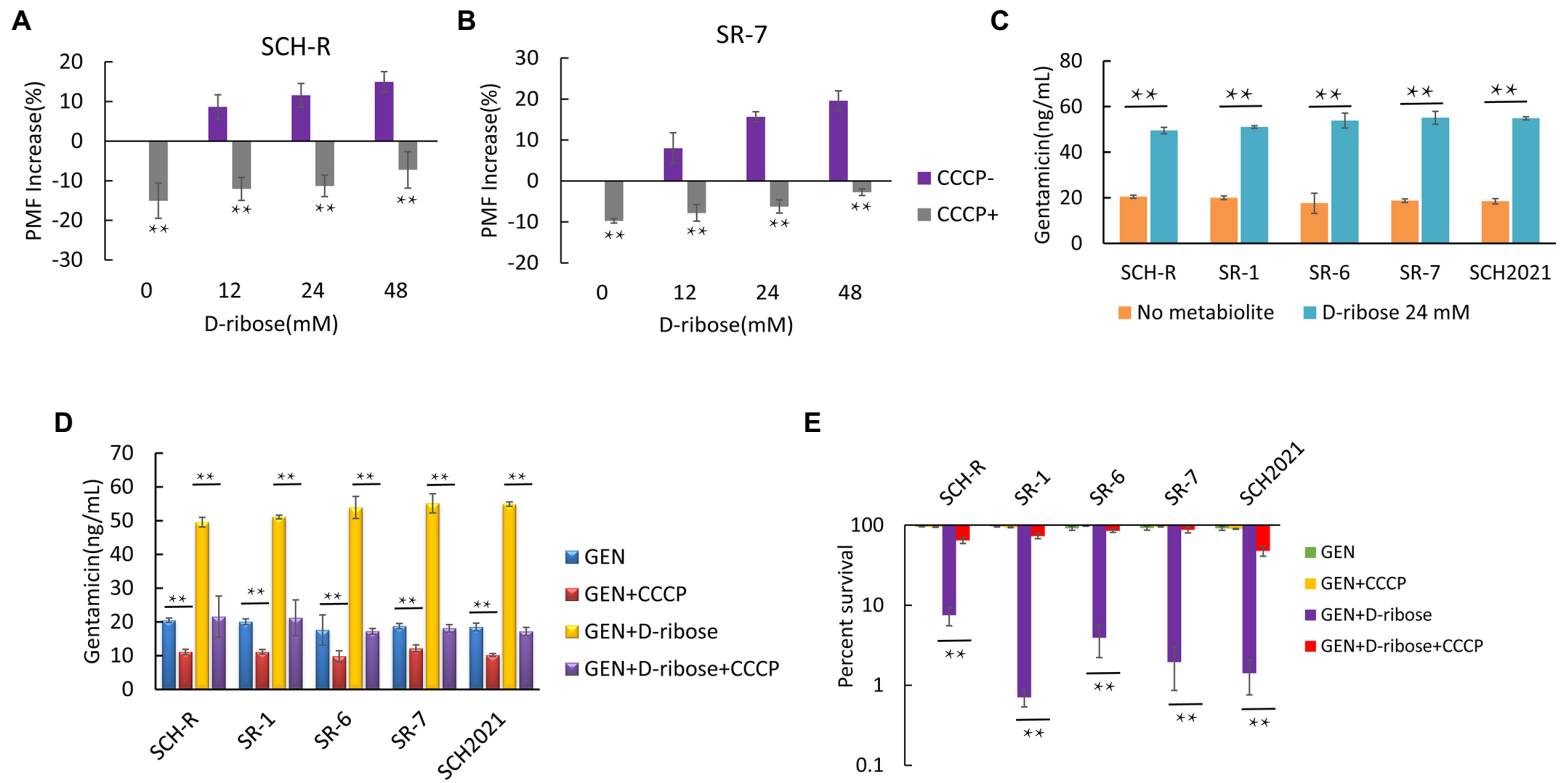
Effect of exogenous D-ribose on membrane potential. **(A)** Variation of PMF in SCH-R by D-ribose dose with or without CCCP. **(B)** Variation of PMF in SR-7 by D-ribose dose with or without CCCP. **(C)** Intracellular concentration of gentamicin in SCH-R and clinical isolates in the presence of 24 mM D-ribose. **(D)** Intracellular concentration of gentamicin in SCH-R and clinical isolates in the presence of 24 mM D-ribose with or without CCCP. GEN, gentamicin. **(E)** Percent survival of SCH-R and clinical isolates in the presence or absence of CCCP and 1 MIC gentamicin plus 24 mM D-ribose. GEN, gentamicin. Results are displayed as the mean ± SEM and three biological repeats are carried out. Significant differences are identified (*p < 0.05 and **p < 0.01).

Glucose increases the PMF of *E. coli* and stimulates the uptake of aminoglycosides and induces cell deaths (Allison et al., 2011). To test whether D-ribose can also enhance the PMF of *Salmonella*, cells were cultured in M9 minimal media with or without D-ribose for 6 h and the PMF was assessed using the *BacLight* bacterial membrane potential Kit. The PMF of SCH-R was increased by 8.62, 11.54, and 14.92% in the presence of 12, 24, and 48 mM D-ribose, respectively (Figure 5A). Similar results were observed using the clinical MDR *Salmonella* SR-7 strain (Figure 5B). To further verify whether elevated PMF can promote drug uptake, an ELISA kit was used to detect the intracellular concentration of gentamicin in the presence of 24 mM D-ribose. The intracellular concentration of gentamicin in SCH-R and clinical *Salmonella* cells increased significantly in the presence of D-ribose (Figure 5C). To verify whether the gentamicin potentiation by D-ribose was result of higher PMF-induced drug uptake and subsequent cell death, PMF was inhibited using the proton ionophore, carbonyl cyanide chlorophenyl hydrazone (CCCP). As expected, CCCP significantly inhibited PMF expression in both the presence and absence of D-ribose (Figures 5A,B), while the intracellular concentration and potentiating effect of gentamicin were declined (Figures 5D,E). These results demonstrated that increased drug uptake induced by D-ribose was responsible for gentamicin killing.

### D-ribose activates the ETC

**Figure 6:**
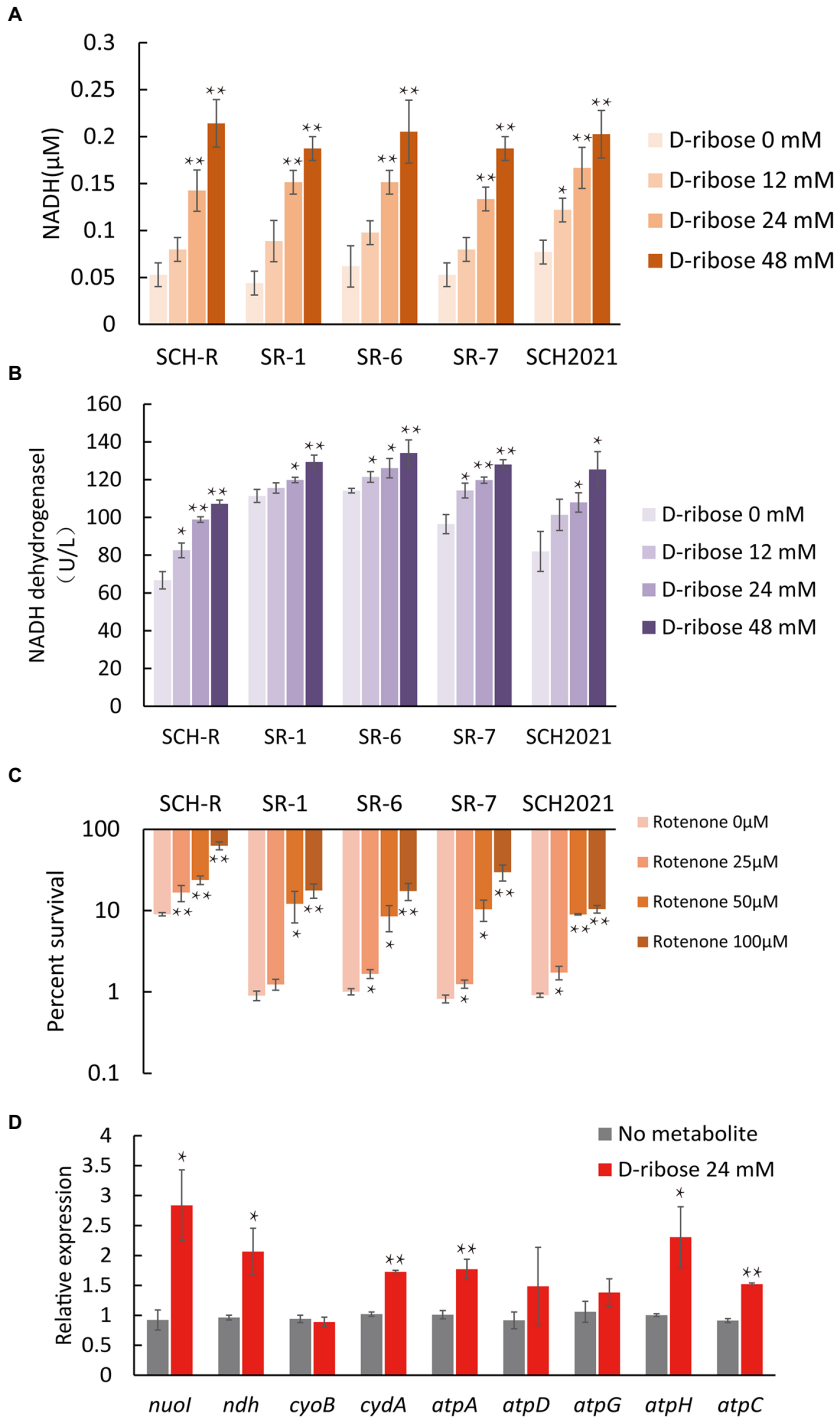
Effect of exogenous D-ribose on the electron transport chain. **(A)** The intracellular NADH concentration of SCH-R and clinical isolates by D-ribose dose. **(B)** Activity of NADH dehydrogenase 1 in SCH-R and clinical isolates by D-ribose dose. **(C)** Percent survival of SCH-R and clinical isolates by rotenone dose and 1 MIC gentamicin plus 24 mM D-ribose. **(D)** QRT-PCR of key electron transport chain genes in the presence 24 mM D-ribose. *nuoI*, NADH dehydrogenase I chain I; *ndh*, NADH:quinone reductase; *cyoB*, cytochrome o ubiquinol oxidase subunit I; *cydA*, cytochrome BD2 subunit I; *atpA*, *atpD*, *atpG*, *atpH* and *atpC*, membrane-bound ATP synthase, F1 sector, alpha-subunit, beta-subunit, gamma-subunit, delta-subunit, and epsilon-subunit. Results are displayed as the mean ± SEM and three biological repeats are carried out. Significant differences are identified (*p < 0.05 and **p < 0.01).

To demonstrate that D-ribose increased PMF by activating ETC. First, the intracellular NADH concentration and NADH dehydrogenase 1 activity of SCH-R were detected. The result showed that both NADH concentration and NADH dehydrogenase 1 activity of SCH-R were elevated by D-ribose in a dose-dependent manner (Figures 6A,B). In addition, rotenone, an ETC inhibitor, abolished D-ribose mediated synergy (Figure 6C). Similar results were obtained using SCH2021 and clinical MDR *Salmonella* (Figures 6A–C). Finally, the expression of ETC-related genes in SCH-R upregulated significantly after adding D-ribose (Figure 6D). These data suggested that D-ribose enhanced PMF by stimulating ETC, and then increased drug uptake, induced cell death ultimately.

### Activated central carbon metabolism drives aminoglycoside-mediated killing

**Figure 7:**
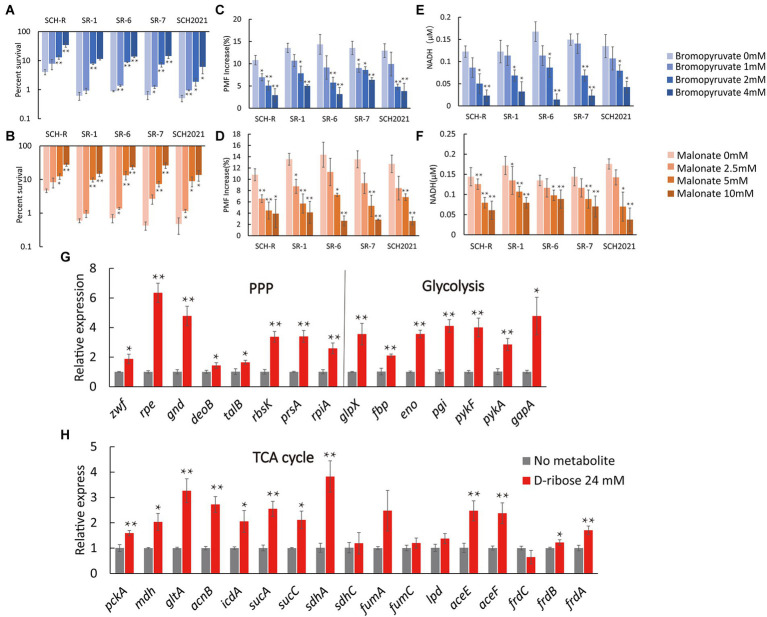
Effect of exogenous D-ribose on central carbon metabolism. **(A,B)** Percent survival of SCH-R and clinical isolates by **(A)** bromopyruvate and **(B)** malonate dose and 1 MIC gentamicin plus 24 mM D-ribose. **(C,D)** PMF of SCH-R and clinical isolates by **(C)** bromopyruvate and **(D)** malonate dose with 24 mM D-ribose. **(E,F)** Intracellular NADH concentration of SCH-R and clinical isolates by **(E)** bromopyruvate and **(F)** malonate dose with 24 mM D-ribose. **(G,H)** QRT-PCR for expression of key central carbon metabolism genes in the presence of 24 mM D-ribose. Results are displayed as the mean ± SEM and three biological repeats are carried out. Significant differences are identified (*p < 0.05 and **p < 0.01).

**Figure 8:**
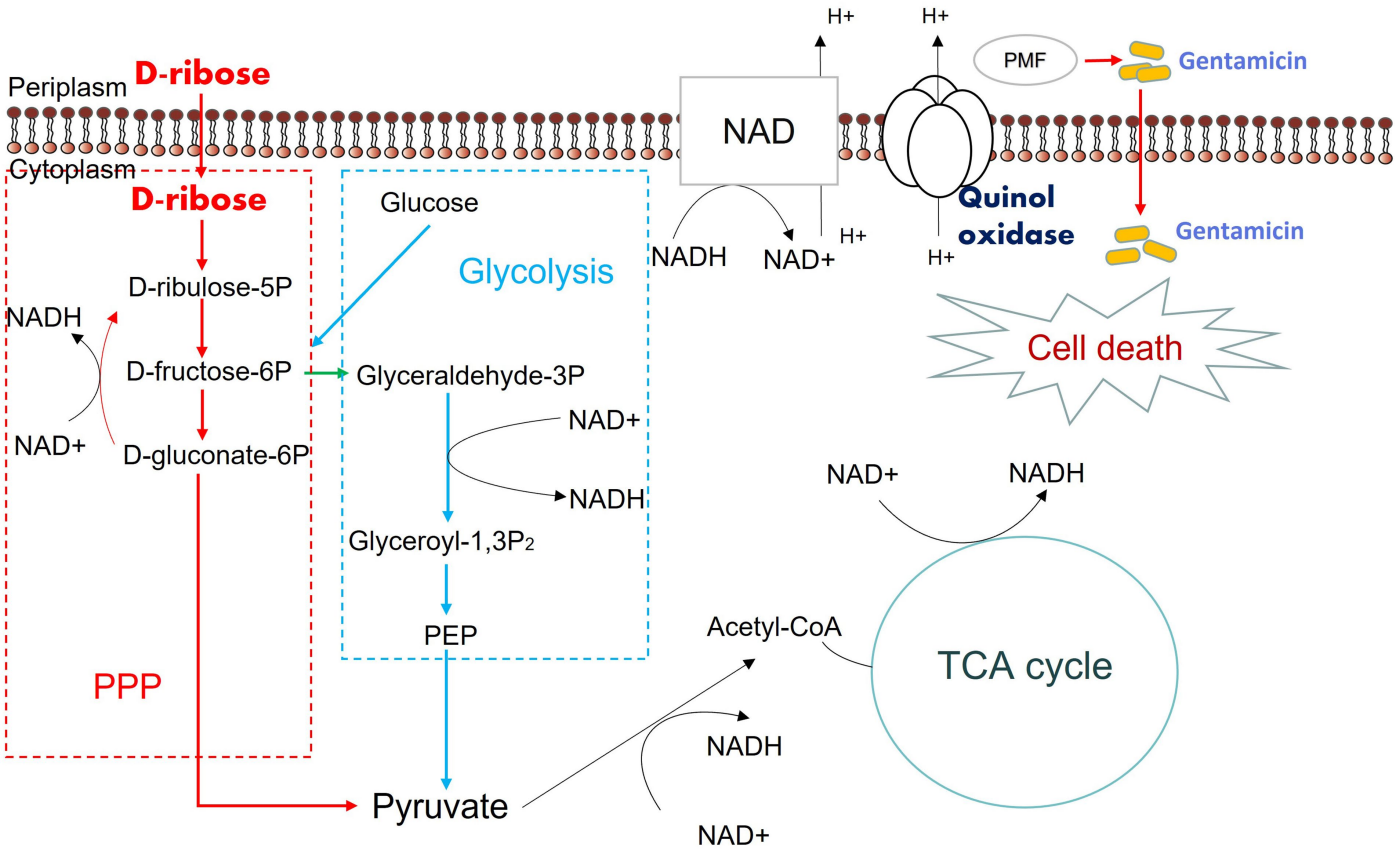
Mechanism of exogenous D-ribose promotes gentamicin treatment of drug-resistant *Salmonella*. D-ribulose 5P, D-ribulose 5-phosphate; D-fructose 6P, D-fructose 6-phosphate; D-gluconate-6P, 6-phospho-D-gluconate; Glyceraldehyde 3P, Glyceraldehyde 3-phosphate; Glyceroyl-1,3P_2_, 3-phospho-D-glyceroyl phosphate; PEP, Phosphoenolpyruvate.

We assumed that D-ribose activated central carbon metabolism to promote ETC polarization. First, two TCA cycle inhibitors, bromopyruvate and malonate, were used to verify this. Specifically, bromopyruvate inhibits the E1 subunit of pyruvate dehydrogenase, malonate competitively inhibits succinate dehydrogenase. Cell survival increased as bromopyruvate and malonate concentrations rose (Figures 7A,B). In addition, lower PMF and NADH levels were detected after adding bromopyruvate or malonate (Figures 7C–F). These findings indicated that TCA inhibition eliminated the D-ribose-mediated potential. Furthermore, most genes involved in central carbon metabolism were upregulated by D-ribose (Figure 7G). This indicated that D-ribose was involved in stimulating the central carbon metabolism of drug-resistant bacteria, producing more NADH, polarizing ETC, increasing PMF, and promoting drug uptake and cell death (Figure 8).

## Discussion

This study characterized the intracellular metabolic profile of gentamicin-sensitive *S.* Choleraesuis, SCH-S, and the homologous gentamicin-resistant strain, SCH-R. A total of 114 metabolites, including amino acids, carbohydrates, nucleotides, and their derivatives, in SCH-R changed significantly relative to SCH-S. The pathway enrichment results of this study are similar to those of Peng et al., who used GC–MS to compare the metabolic profiles of kanamycin-sensitive and drug-resistant *Edwardsiella tarda* (Peng et al., 2015b). It is important to note that drug-resistant *S.* Choleraesuis showed broader metabolic fluctuations and enrichment of more metabolic pathways. This may be related to differences between the bacterial species or because more metabolites were identified in the current study.

Results indicated that oxidative phosphorylation was the most impacted pathway. While ATP and ADP levels were diminished, nicotinamide adenine dinucleotide (NAD^+^) production was increased. Oxidative phosphorylation, the pathway by which bacteria produce ATP, involves a series of chemical reactions in the ETC, and an activated ETC is necessary for PMF (Gao et al., 2019). Importantly, PMF is essential for aminoglycoside internalization (Taber et al., 1987). In SCH-R, the reduced production of ATP and ADP and increased production of NAD^+^, indicated that the PMF of SCH-R was affected, a finding confirmed by later experiments (Figure 3B). Carbohydrate metabolism, which involves glycolysis/gluconeogenesis, the pentose phosphate pathway, fructose and mannose metabolism, and pentose and gluconate interconversion, was also seriously disrupted in SCH-R. All PPP metabolites were down-regulated, of which D-ribose, was the most inhibited. Central carbon metabolism, involving glycolysis, the pentose phosphate pathway, and the TCA cycle, is the core component of cell metabolism, responsible for converting nutrients into biomass to maintain life (Baughn and Rhee, 2014; Barbier et al., 2018; Dolan et al., 2020). Disrupted central carbon metabolism and energy metabolism result in drug inactivation in bacterial cells (Chen et al., 2022). While the TCA cycle was not affected in SCH-R, the fluctuation of amino acid metabolism and other metabolic pathways, as well as the qPCR results, suggested that the TCA cycle was disrupted. These results suggest that bacteria may require central carbon metabolism and energy metabolism to acquire resistance.

A recent study confirmed that activated purine metabolism could restore the sensitivity of multidrug-resistant bacteria to antibiotics. This may be due to increased purine metabolism, which activates the CpxA/CpxR two-component system, promotes OmpF expression, and induces drug uptake (Zhao et al., 2021). Coincidentally，both purine and pyrimidine metabolism were seriously impacted in SCH-R and the pentose phosphate pathway and alanine, aspartate, and glutamate metabolism were shown to directly affect purine metabolism through 5-phosphoribosyl diphosphate and 5-phosphoribosylamine, respectively. Furthermore, it is worth noting that nutrients can also contribute to riboflavin metabolism through the pentose phosphate pathway, leading to ROS production, a cause of bacterial death induced by bactericidal antibiotics (Kohanski et al., 2007; Chen et al., 2022). Whether purine and pyrimidine metabolism are involved in the inactivation of gentamicin, and whether there is a certain co-action between them and central carbon metabolism mediating the resistance of SCH-R to gentamicin remains to be further studied. Meanwhile, disrupted central carbon metabolism and increased fatty acid synthesis mediate ceftazidime and ciprofloxacin resistance jointly (Liu et al., 2019; Su et al., 2021). While fatty acid synthesis was not enriched in SCH-R, glycerolipid and glycerophospholipid metabolism indirectly reflect the impact on fatty acid synthesis. These results suggested that SCH-R acquires gentamicin resistance through a universal metabolic shift, including upstream amino acid metabolism, central carbon metabolism at the core, and downstream metabolism including nucleotide metabolism and fatty acid synthesis. Thus, it is evident that fluctuations in central carbon metabolism, accompanied by changes in other metabolic pathways, are critical for drug inactivation.

Antibiotic efficacy is enhanced by altering the metabolic state of bacteria (Allison et al., 2011). Importantly, repressing metabolites through exogenous supplementation can alter the sensitivity of resistant strains (Peng et al., 2015a). Such a strategy was first implemented in resistant *Escherichia coli* and *Staphylococcus aureus* (Allison et al., 2011). This study found that glucose, fructose, and mannitol can promote glycolysis, stimulate the ETC, induce PMF, promote gentamicin uptake, and eliminate *E. coli* persister cells and *Staphylococcus aureus* biofilm. Metabolite-dependent aminoglycoside potentiation has also been applied to aminoglycoside-resistant bacteria, such as *Edwardsiella tarda* (Su et al., 2015), *Vibrio alginolyticus* (Li et al., 2020), and *Pseudomonas aeruginosa* (Meylan et al., 2017). Thus, it was probable that metabolome-reprogramming could also enhance the effect of antibiotics against SCH-R and other drug-resistant *Salmonella*. Indeed, D-ribose was shown to significantly enhance the gentamicin-mediated killing of both SCH-R and multidrug-resistant *S.* Choleraesuis, *S.* Derby, and *S.* Typhimurium. Allison et al. (2011) found that metabolites enter metabolism through glycolysis rather than the pentose phosphate pathway, inducing rapid gentamicin killing of persisters. However, D-ribose, which enters metabolism *via* the pentose phosphate pathway, also promotes the gentamicin-mediated killing of drug-resistance *Salmonella*. Notably, glucose, fructose, and mannitol failed to increase the effect of aminoglycosides against SCH-R and multidrug-resistant *Salmonella* (data not shown), but are shown to enhance the impact of aminoglycosides on *Escherichia coli*, *Staphylococcus aureus*, *Pseudomonas aeruginosa*, and *Edwardsiella tarda* (Peng et al., 2015b). This finding suggests that the strengthening effect of D-ribose may target drug-resistant bacteria rather than persisters.

The current study demonstrated that D-ribose significantly increased the gentamicin-, tobramycin-, and apramycin-mediated killing of *S.* Choleraesuis, *S.* Derby, and *S.* Typhimurium (Figures 4A–G). D-ribose does not have the same impact on kanamycin and spectinomycin (Supplementary Figures S2A,B), however, which may be explained by the higher MIC of these drugs in the wild strains (Table 1). Alternatively, this mechanism of metabolite-mediated killing may be used by bactericidal antibiotics but not bacteriostatic drugs (Allison et al., 2011; Peng et al., 2015b; Meylan et al., 2017). Whether there are additional factors that prevent D-ribose from impacting kanamycin and spectinomycin requires further investigation. Interestingly, in the presence of 1 MIC gentamicin and 24 mM D-ribose, the percent survival of SCH-R was significantly higher than the clinical isolates (Supplementary Figure S3A). As illustrated by Su et al. (2015), this may be attributable to the weaker growth capacity of SCH-R than the clinical isolates and SCH-S after 6 h (Supplementary Figures S3B,C). Additional studies are needed to determine whether this is common among lab and clinical strains or whether it occurs sporadically.

Increased central carbon metabolism, especially the TCA cycle, is the core of metabolome reprogramming. Our previous work confirmed that citrulline and glutamine enhanced the apramycin-mediated killing of *Salmonella via* the TCA cycle (Yong et al., 2021). In the current study, D-ribose significantly increased central carbon metabolism. D-ribose promoted the uptake of drugs by drug-resistant bacteria, which is attributed to the elevated PMF maintained by the ETC. Importantly, CCCP and rotenone significantly increased cell survival. Exogenous D-ribose also stimulated NADH and NADH dehydrogenase and promoted central carbon metabolism, an important source of NADH. These findings suggested that D-ribose-induced central carbon metabolism is critical for gentamicin potentiation. While D-ribose indirectly affects other metabolic pathways, such as purine metabolism and riboflavin metabolism, the effect of D-ribose on these pathways requires further exploration.

In summary, we utilized a metabolomics approach to explore the metabolic mechanism of gentamicin resistance in *S.* Choleraesuis. Disturbed central carbon metabolism, accompanied by fluctuations in metabolic pathways such as amino acid, nucleotide, vitamin, and cofactor metabolism, were identified as characteristics of gentamicin-resistant *S.* Choleraesuis. In addition, D-ribose, which can promote the effect of gentamicin on multidrug-resistant *Salmonella*, was identified as a potential new antibiotic adjuvant. We confirmed that D-ribose promoted the central carbon metabolism of drug-resistant bacteria, increasing glycolysis, the pentose phosphate pathway and TCA cycle, NADH production, ETC activation, PMF, and subsequent drug uptake and cell death. This study characterizes the metabolic mechanism of gentamicin-resistant *Salmonella* and provides evidence that D-ribose can serve as a potential adjuvant to enhance the efficacy of aminoglycosides.

## Data availability statement

The original contributions presented in the study are included in the article/Supplementary material, further inquiries can be directed to the corresponding author.

## Author contributions

YZ carried out the main experiments and data analysis and wrote the manuscript. YY and CZ participated in the *in vitro* validation tests. HY participated in the isolation and identification of clinical drug-resistant bacteria. BF conceived and designed the experiments. All authors contributed to the article and approved the submitted version.

## Funding

This work was funded by the Local Innovative and Research Teams Project of Guangdong Pearl River Talents Program (No. 2019BT02N054).

## Conflict of interest

YY is employed by Guangdong Wens Dahuanong Biotechnology Limited Company.

The remaining authors declare that the research was conducted in the absence of any commercial or financial relationships that could be construed as a potential conflict of interest.

## Publisher’s note

All claims expressed in this article are solely those of the authors and do not necessarily represent those of their affiliated organizations, or those of the publisher, the editors and the reviewers. Any product that may be evaluated in this article, or claim that may be made by its manufacturer, is not guaranteed or endorsed by the publisher.

## References

[ref1] Allison K. R. Brynildsen M. P. Collins J. J. (2011). Metabolite-enabled eradication of bacterial persisters by aminoglycosides. Nature 473, 216–220. doi: 10.1038/nature10069, PMID: 21562562 PMC3145328

[ref2] Bald D. Villellas C. Lu P. Koul A. (2017). Targeting energy metabolism in mycobacterium tuberculosis, a new paradigm in Antimycobacterial drug discovery. MBio 8, e00272–e00217. doi: 10.1128/mBio.00272-17 28400527 PMC5388804

[ref3] Barbier T. Zuniga-Ripa A. Moussa S. Plovier H. Sternon J. F. Lazaro-Anton L.. (2018). Brucella central carbon metabolism: an update. Crit. Rev. Microbiol. 44, 182–211. doi: 10.1080/1040841X.2017.1332002 28604247

[ref4] Baughn A. D. Rhee K. Y. (2014). Metabolomics of central carbon metabolism in mycobacterium tuberculosis. Microbiol. Spectr. 2:2.3.02. doi: 10.1128/microbiolspec.MGM2-0026-2013, PMID: 26103978

[ref5] Bottger E. C. Crich D. (2020). Aminoglycosides: time for the resurrection of a neglected class of Antibacterials? ACS Infect. Dis. 6, 168–172. doi: 10.1021/acsinfecdis.9b00441, PMID: 31855407 PMC7024022

[ref6] Breijyeh Z. Jubeh B. Karaman R. (2020). Resistance of gram-negative bacteria to current antibacterial agents and approaches to resolve it. Molecules 25:1340. doi: 10.3390/molecules25061340, PMID: 32187986 PMC7144564

[ref7] Bush K. Courvalin P. Dantas G. Davies J. Eisenstein B. Huovinen P.. (2011). Tackling antibiotic resistance. Nat. Rev. Microbiol. 9, 894–896. doi: 10.1038/nrmicro2693, PMID: 22048738 PMC4206945

[ref8] Castanheira M. Simner P. J. Bradford P. A. (2021). Extended-spectrum beta-lactamases: an update on their characteristics, epidemiology and detection. JAC Antimicrob. Resist. 3:dlab092. doi: 10.1093/jacamr/dlab092, PMID: 34286272 PMC8284625

[ref9] Castro-Santos P. Laborde C. M. Diaz-Pena R. (2015). Genomics, proteomics and metabolomics: their emerging roles in the discovery and validation of rheumatoid arthritis biomarkers. Clin. Exp. Rheumatol. 33, 279–286. 25572119

[ref10] Castro-Vargas R. E. Herrera-Sanchez M. P. Rodriguez-Hernandez R. Rondon-Barragan I. S. (2020). Antibiotic resistance in salmonella spp. isolated from poultry: a global overview. Vet. World 13, 2070–2084. doi: 10.14202/vetworld.2020.2070-2084, PMID: 33281339 PMC7704309

[ref11] CDC (2019). Antibiotic resistance threats in the United States [online]. Available at: https://www.cdc.gov/drugresistance/pdf/threats-report/2019-ar-threats-report-508.pdf (Accessed July 8, 2022).

[ref12] Chang C. C. Lin Y. H. Chang C. F. Yeh K. S. Chiu C. H. Chu C.. (2005). Epidemiologic relationship between fluoroquinolone-resistant salmonella enterica Serovar Choleraesuis strains isolated from humans and pigs in Taiwan (1997 to 2002). J. Clin. Microbiol. 43, 2798–2804. doi: 10.1128/JCM.43.6.2798-2804.2005, PMID: 15956400 PMC1151913

[ref13] Chen Y. T. Yang K. X. Dai Z. Y. Yi H. Peng X. X. Li H.. (2022). Repressed central carbon metabolism and its effect on related metabolic pathways in Cefoperazone/Sulbactam-resistant Pseudomonas aeruginosa. Front. Microbiol. 13:847634. doi: 10.3389/fmicb.2022.847634, PMID: 35308347 PMC8927769

[ref14] Cheng Z. X. Gong Q. Y. Wang Z. Chen Z. G. Ye J. Z. Li J.. (2017). Edwardsiella tarda tunes Tricarboxylic acid cycle to evade complement-mediated killing. Front. Immunol. 8:1706. doi: 10.3389/fimmu.2017.01706, PMID: 29270172 PMC5725468

[ref15] Chiu C. H. Su L. H. Chu C. (2004). Salmonella enterica serotype Choleraesuis: epidemiology, pathogenesis, clinical disease, and treatment. Clin. Microbiol. Rev. 17, 311–322. doi: 10.1128/CMR.17.2.311-322.2004, PMID: 15084503 PMC387403

[ref16] Ciorba V. Odone A. Veronesi L. Pasquarella C. Signorelli C. (2015). Antibiotic resistance as a major public health concern: epidemiology and economic impact. Ann. Ig. 27, 562–579. doi: 10.7416/ai.2015.2048, PMID: 26152543

[ref17] Cohen J. I. Bartlett J. A. Corey G. R. (1987). Extra-intestinal manifestations of salmonella infections. Medicine (Baltimore) 66, 349–388. doi: 10.1097/00005792-198709000-00003 3306260

[ref18] Dolan S. K. Kohlstedt M. Trigg S. Vallejo Ramirez P. Kaminski C. F. Wittmann C.. (2020). Contextual flexibility in Pseudomonas aeruginosa central carbon metabolism during growth in single carbon sources. MBio 11, e02684–e02619. doi: 10.1128/mBio.02684-19 32184246 PMC7078475

[ref19] Ernholm L. Sternberg-Lewerin S. Agren E. Stahl K. Hulten C. (2022). First detection of salmonella enterica Serovar Choleraesuis in free ranging European wild boar in Sweden. Pathogens 11:723. doi: 10.3390/pathogens11070723, PMID: 35889969 PMC9324790

[ref20] Fisher R. A. Gollan B. Helaine S. (2017). Persistent bacterial infections and persister cells. Nat. Rev. Microbiol. 15, 453–464. doi: 10.1038/nrmicro.2017.42 28529326

[ref21] Gao M. Yi J. Zhu J. Minikes A. M. Monian P. Thompson C. B.. (2019). Role of mitochondria in Ferroptosis. Mol. Cell 73, 354–363.e3. doi: 10.1016/j.molcel.2018.10.042, PMID: 30581146 PMC6338496

[ref22] Gil Molino M. Risco Perez D. Goncalves Blanco P. Fernandez Llario P. Quesada Molina A. Garcia Sanchez A.. (2019). Outbreaks of antimicrobial resistant salmonella Choleraesuis in wild boars piglets from Central-Western Spain. Transbound. Emerg. Dis. 66, 225–233. doi: 10.1111/tbed.13003, PMID: 30144295 PMC7168558

[ref23] Hendriksen R. S. Vieira A. R. Karlsmose S. Wong L. F. Danilo M. Jensen A. B.. (2011). Global monitoring of salmonella serovar distribution from the World Health Organization global foodborne infections network country data Bank: results of quality assured laboratories from 2001 to 2007. Foodborne Pathog. Dis. 8, 887–900. doi: 10.1089/fpd.2010.0787, PMID: 21492021

[ref24] Hogberg L. D. Heddini A. Cars O. (2010). The global need for effective antibiotics: challenges and recent advances. Trends Pharmacol. Sci. 31, 509–515. doi: 10.1016/j.tips.2010.08.002, PMID: 20843562

[ref25] Johnson C. H. Ivanisevic J. Siuzdak G. (2016). Metabolomics: beyond biomarkers and towards mechanisms. Nat. Rev. Mol. Cell Biol. 17, 451–459. doi: 10.1038/nrm.2016.25, PMID: 26979502 PMC5729912

[ref26] Kohanski M. A. Dwyer D. J. Hayete B. Lawrence C. A. Collins J. J. (2007). A common mechanism of cellular death induced by bactericidal antibiotics. Cells 130, 797–810. doi: 10.1016/j.cell.2007.06.049, PMID: 17803904

[ref27] Kok M. Maton L. van der Peet M. Hankemeier T. van Hasselt J. G. C. (2022). Unraveling antimicrobial resistance using metabolomics. Drug Discov. Today 27, 1774–1783. doi: 10.1016/j.drudis.2022.03.015, PMID: 35341988

[ref28] Kovac J. R. Pastuszak A. W. Lamb D. J. (2013). The use of genomics, proteomics, and metabolomics in identifying biomarkers of male infertility. Fertil. Steril. 99, 998–1007. doi: 10.1016/j.fertnstert.2013.01.111, PMID: 23415969 PMC3652543

[ref29] Li L. Su Y. B. Peng B. Peng X. X. Li H. (2020). Metabolic mechanism of colistin resistance and its reverting in vibrio alginolyticus. Environ. Microbiol. 22, 4295–4313. doi: 10.1111/1462-2920.15021, PMID: 32291842

[ref30] Li H. Wang Y. Meng Q. Wang Y. Xia G. Xia X.. (2019). Comprehensive proteomic and metabolomic profiling of mcr-1-mediated colistin resistance in Escherichia coli. Int. J. Antimicrob. Agents 53, 795–804. doi: 10.1016/j.ijantimicag.2019.02.014, PMID: 30811973

[ref31] Liu S. R. Peng X. X. Li H. (2019). Metabolic mechanism of ceftazidime resistance in vibrio alginolyticus. Infect Drug Resist. 12, 417–429. doi: 10.2147/IDR.S179639, PMID: 30863124 PMC6388739

[ref32] Longo A. Losasso C. Vitulano F. Mastrorilli E. Turchetto S. Petrin S.. (2019). Insight into an outbreak of salmonella Choleraesuis var. Kunzendorf in wild boars. Vet Microbiol 238:108423. doi: 10.1016/j.vetmic.2019.108423, PMID: 31648730

[ref33] Luk-In S. Chatsuwan T. Pulsrikarn C. Bangtrakulnonth A. Rirerm U. Kulwichit W. (2018). High prevalence of ceftriaxone resistance among invasive salmonella enterica serotype Choleraesuis isolates in Thailand: the emergence and increase of CTX-M-55 in ciprofloxacin-resistant S. Choleraesuis isolates. Int J Med Microbiol 308, 447–453. doi: 10.1016/j.ijmm.2018.03.008, PMID: 29605531

[ref34] Mahon B. E. Fields P. I. (2016). Invasive infections with Nontyphoidal salmonella in sub-Saharan Africa. Microbiol Spectr 4:4.3.18. doi: 10.1128/microbiolspec.EI10-0015-2016 27337467

[ref35] Meylan S. Porter C. B. M. Yang J. H. Belenky P. Gutierrez A. Lobritz M. A.. (2017). Carbon sources tune antibiotic susceptibility in Pseudomonas aeruginosa via Tricarboxylic acid cycle control. Cell Chem Biol 24, 195–206. doi: 10.1016/j.chembiol.2016.12.015, PMID: 28111098 PMC5426816

[ref36] Molino G. M. Garcia A. Zurita S. G. Martin-Cano F. E. Garcia-Jimenez W. Risco D.. (2020). Spread of antimicrobial resistance by salmonella enterica Serovar Choleraesuis between close domestic and wild environments. Antibiotics (Basel) 9:750. doi: 10.3390/antibiotics9110750, PMID: 33137987 PMC7692705

[ref37] Oransathid W. Sukhchat P. Margulieux K. Wongpatcharamongkol N. Kormanee R. Pimsawat T.. (2022). First report: Colistin resistance gene mcr-3.1 in salmonella enterica serotype Choleraesuis isolated from human blood sample from Thailand. Microb. Drug Resist. 28, 102–105. doi: 10.1089/mdr.2020.0553, PMID: 34242096

[ref38] Otto M. (2013). Community-associated MRSA: what makes them special? Int. J. Med. Microbiol. 303, 324–330. doi: 10.1016/j.ijmm.2013.02.007, PMID: 23517691 PMC3729626

[ref39] Papic B. Kusar D. Micunovic J. Vidrih S. Pirs M. Ocepek M.. (2021). Genomic insights into salmonella Choleraesuis var. Kunzendorf outbreak reveal possible interspecies transmission. Vet. Microbiol. 263:109282. doi: 10.1016/j.vetmic.2021.109282, PMID: 34785475

[ref40] Peng B. Li H. Peng X. X. (2015a). Functional metabolomics: from biomarker discovery to metabolome reprogramming. Protein Cell 6, 628–637. doi: 10.1007/s13238-015-0185-x, PMID: 26135925 PMC4537470

[ref41] Peng B. Su Y. B. Li H. Han Y. Guo C. Tian Y. M.. (2015b). Exogenous alanine and/or glucose plus kanamycin kills antibiotic-resistant bacteria. Cell Metab. 21, 249–262. doi: 10.1016/j.cmet.2015.01.008 25651179

[ref42] Poulikakos P. Falagas M. E. (2013). Aminoglycoside therapy in infectious diseases. Expert. Opin. Pharmacother. 14, 1585–1597. doi: 10.1517/14656566.2013.806486 23746121

[ref43] Provenzani A. Hospodar A. R. Meyer A. L. Leonardi Vinci D. Hwang E. Y. Butrus C. M.. (2020). Multidrug-resistant gram-negative organisms: a review of recently approved antibiotics and novel pipeline agents. Int. J. Clin. Pharm. 42, 1016–1025. doi: 10.1007/s11096-020-01089-y, PMID: 32638294

[ref44] Reyes K. Bardossy A. C. Zervos M. (2016). Vancomycin-resistant enterococci: epidemiology, infection prevention, and control. Infect. Dis. Clin. N. Am. 30, 953–965. doi: 10.1016/j.idc.2016.07.009 27660091

[ref45] Ryan D. G. Yang M. Prag H. A. Blanco G. R. Nikitopoulou E. Segarra-Mondejar M.. (2021). Disruption of the TCA cycle reveals an ATF4-dependent integration of redox and amino acid metabolism. elife 10:e72593. doi: 10.7554/eLife.72593, PMID: 34939929 PMC8735863

[ref46] Stokes J. M. Lopatkin A. J. Lobritz M. A. Collins J. J. (2019). Bacterial metabolism and antibiotic efficacy. Cell Metab. 30, 251–259. doi: 10.1016/j.cmet.2019.06.009, PMID: 31279676 PMC6990394

[ref47] Su Y. B. Kuang S. F. Ye J. Z. Tao J. J. Li H. Peng X. X.. (2021). Enhanced biosynthesis of fatty acids is associated with the Acquisition of Ciprofloxacin Resistance in Edwardsiella tarda. mSystems 6:e0069421. doi: 10.1128/mSystems.00694-21 34427511 PMC8407472

[ref48] Su Y. B. Peng B. Han Y. Li H. Peng X. X. (2015). Fructose restores susceptibility of multidrug-resistant Edwardsiella tarda to kanamycin. J. Proteome Res. 14, 1612–1620. doi: 10.1021/pr501285f, PMID: 25675328

[ref49] Taber H. W. Mueller J. P. Miller P. F. Arrow A. S. (1987). Bacterial uptake of aminoglycoside antibiotics. Microbiol. Rev. 51, 439–457. doi: 10.1128/mr.51.4.439-457.1987, PMID: 3325794 PMC373126

[ref50] Touati A. (2019). Aminoglycoside resistance mechanism inference algorithm: implication for underlying resistance mechanisms to aminoglycosides. EBioMedicine 46:8. doi: 10.1016/j.ebiom.2019.07.045, PMID: 31350220 PMC6712273

[ref51] Tyson G. H. Li C. Harrison L. B. Martin G. Hsu C. H. Tate H.. (2021). A multidrug-resistant salmonella Infantis clone is spreading and recombining in the United States. Microb. Drug Resist. 27, 792–799. doi: 10.1089/mdr.2020.0389, PMID: 33232624 PMC11555764

[ref52] WHO (2017). List of critically important antimicrobials for human medicine [online]. Available at: https://apps.who.int/iris/bitstream/handle/10665/255027/9789241512220-eng.pdf?sequence=1 (Accessed 21 August, 2022).

[ref53] Yang C. C. Chuang F. R. Wu C. H. Chen J. B. Lee C. H. Lee C. T. (2012). Refractory salmonella enterica serotype Choleraesuis-related renal cyst infection in a patient with autosomal dominant polycystic kidney disease undergoing hemodialysis treated successfully with intracystic ciprofloxacin infusion. Med. Princ. Pract. 21, 576–578. doi: 10.1159/000339199, PMID: 22710499

[ref54] Yin W. Wang Y. Liu L. He J. (2019). Biofilms: the microbial "protective clothing" in extreme environments. Int. J. Mol. Sci. 20:3423. doi: 10.3390/ijms20143423, PMID: 31336824 PMC6679078

[ref55] Yong Y. Zhou Y. Liu K. Liu G. Wu L. Fang B. (2021). Exogenous Citrulline and glutamine contribute to reverse the resistance of salmonella to Apramycin. Front. Microbiol. 12:759170. doi: 10.3389/fmicb.2021.759170, PMID: 34721368 PMC8552007

[ref56] Zhao X. L. Chen Z. G. Yang T. C. Jiang M. Wang J. Cheng Z. X.. (2021). Glutamine promotes antibiotic uptake to kill multidrug-resistant uropathogenic bacteria. Sci. Transl. Med. 13:eabj0716. doi: 10.1126/scitranslmed.abj0716, PMID: 34936385

