# Supplementary material for: Exogenous D-ribose promotes gentamicin treatment of several drug-resistant Salmonella
Source: Front Microbiol. 2022 Nov 7;13:1053330. doi: 10.3389/fmicb.2022.1053330 (PMC9676500; doi:10.3389/fmicb.2022.1053330)
Supplement: Supplementary file 2 [file Data_Sheet_2.docx]

Supplementary Material

# Supplementary Figures

#
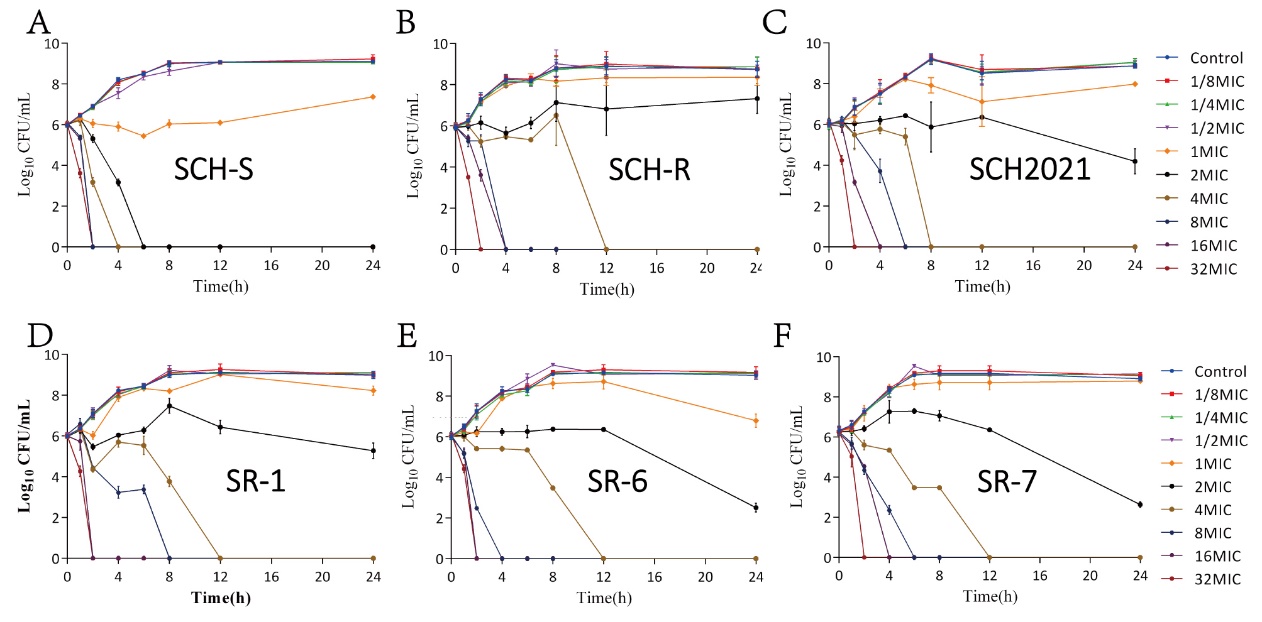


# Supplementary Figure S1. In *vitro* gentamicin-exposed growth curve of SCH-S(A), SCH-R(B), SR-1(C), SR-6(D), SR-7(E), SCH2021(F).


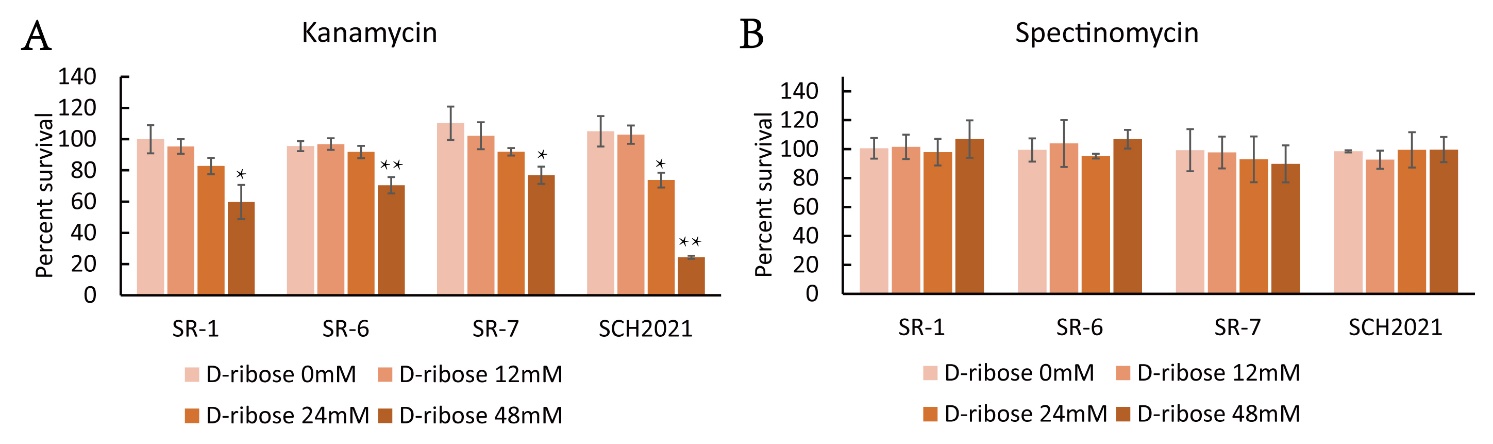


**Supplementary Figure S2.** **Effect of exogenous D-ribose on clinical isolates of drug-resistant *Salmonella*.**

A. Percent survival of SCH-R in the presence of 1 MIC kanamycin by D-ribose dose.

B. Percent survival of SCH-R in the presence of 1 MIC spectinomycin by D-ribose dose.

Results are displayed as the mean ± SEM and three biological repeats are carried out. Significant differences are identified (**p* < 0.05 and ***p* < 0.01).


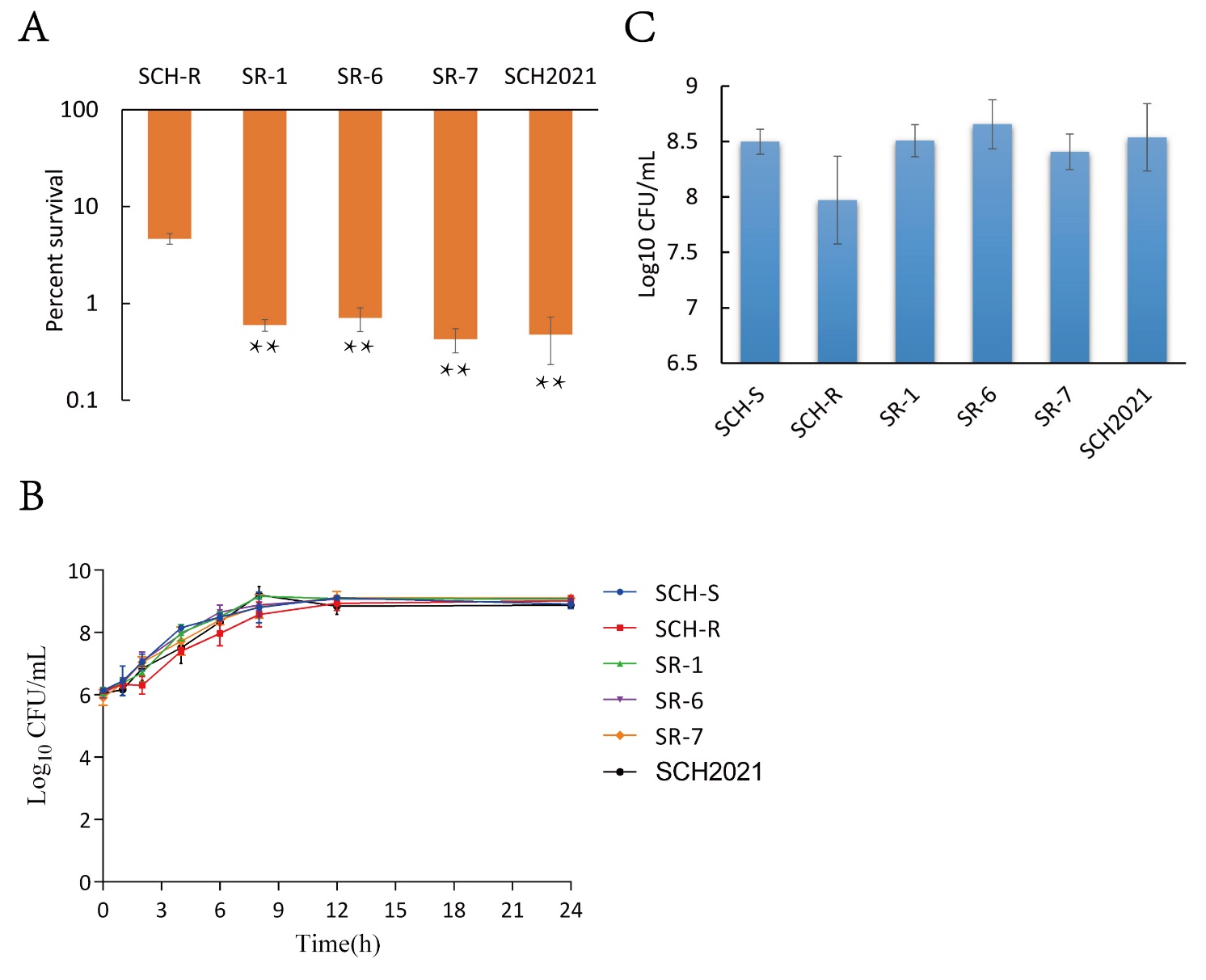


**Supplementary Figure S3.** **Growth curve of lab stains and clinical isolates.**

1. Percent survival of SCH-R and clinical isolates in the presence of 1MIC gentamicin plus 24 mM D-ribose.
2. Growth curve of lab stains and clinical isolates.
3. Cell concentration (Log_10_ CFU/mL) of all strains at 6 h.

Results are displayed as the mean ± SEM and three biological repeats are carried out. Significant differences are identified (**p* < 0.05 and ***p* < 0.01).
